# Expression of the glucagon-like peptide-1 receptor and its role in regulating autophagy in endometrial cancer

**DOI:** 10.1186/s12885-018-4570-8

**Published:** 2018-06-15

**Authors:** Ranka Kanda, Haruko Hiraike, Osamu Wada-Hiraike, Takayuki Ichinose, Kazunori Nagasaka, Yuko Sasajima, Eiji Ryo, Tomoyuki Fujii, Yutaka Osuga, Takuya Ayabe

**Affiliations:** 1 Department of Obstetrics and Gynecology, Teikyo University School of Medicine, 2-11-1, Kaga, Itabashi-ku, Tokyo, 173 0003 Japan; 2 Department of Obstetrics and Gynecology, The University of Tokyo, Tokyo, Japan; 3 Department of Pathology, Teikyo University School of Medicine, Tokyo, Japan

**Keywords:** Glucagon-like peptide-1 receptor, Endometrial cancer, Autophagy, AMPK, Progression-free survival

## Abstract

**Background:**

A previous report showed that a glucagon-like peptide-1 receptor (GLP-1R) agonist (exenatide) induced apoptosis in endometrial cancer cells. However, the pathophysiological role of GLP-1R in endometrial cancer has not been fully elucidated. Here, we investigated the effects of the GLP-1R agonist liraglutide in endometrial cancer cells and examined the association between GLP-1R expression and clinicopathological characteristics in endometrial cancer patients.

**Methods:**

Human Ishikawa endometrial cancer cells were treated with different concentrations of liraglutide. To assess the effects of liraglutide, cell viability, colony formation, flow cytometry, Western blotting, and immunofluorescence assays were performed. Autophagy induction was examined by analyzing LC3 and p62 expression and autophagosome accumulation. Moreover, using a tissue microarray, we analyzed GLP-1R expression in 154 endometrial cancer tissue samples by immunohistochemistry.

**Results:**

In accordance with the previous report, liraglutide inhibited Ishikawa cell growth in a dose-dependent manner. Liraglutide significantly induced autophagy, and phosphorylated AMPK expression was elevated. Immunohistochemical analysis revealed that GLP-1R expression was associated with positive estrogen receptor and progesterone receptor status, and higher GLP-1R expression was significantly correlated with better progression-free survival.

**Conclusions:**

The use of liraglutide to target autophagy in endometrial cancer cells may be a novel potential treatment for endometrial cancer. Furthermore, higher GLP-1R expression may be associated with better prognosis in endometrial cancer patients.

**Electronic supplementary material:**

The online version of this article (10.1186/s12885-018-4570-8) contains supplementary material, which is available to authorized users.

## Background

Endometrial cancer is one of the most common gynecologic malignancies in developed countries, and its incidence has been increasing in Japan [1, 2].

Endometrial cancer can be classified as type I or type II according to clinical and pathological characteristics [3]. Type I endometrial cancer is associated with an unopposed estrogenic state and low histological grade and typically follows a favorable course, while type II endometrial cancer is not associated with a hyperestrogenic status and is correlated with poorer differentiation. Type I endometrial cancer is strongly linked to obesity [4]. By contrast, type II usually occurs in non-obese patients.

Recently, obesity and type 2 diabetes have been found to be associated with increased endometrial cancer risk and adverse prognosis among endometrial cancer patients, suggesting that insulin resistance is involved in the development of endometrial cancer [5, 6]. Epidemiological and clinical data suggest that metformin, an anti-diabetes drug, could prevent certain cancers, including endometrial cancer. Therefore, metformin is considered to be a promising treatment modality for endometrial cancer. The physiological function of metformin is linked to the activation of AMP-activated protein kinase (AMPK) and suppression of PI3K (phosphatidylinositol-3 kinase)-mTOR (mammalian target of rapamycin) [7], and loss of AMPK activity can promote oncogenesis [8]. In addition, insulin is understood to play an important role in increasing the risk of cancer by activating the insulin growth factor-1 receptor (IGF-1R)/PI3K/Akt/ mTOR signaling pathway [9, 10].

Intestinal peptides that regulate blood glucose level include glucose-dependent insulinotropic polypeptide (GIP) secreted from proximal small intestine and glucagon-like peptide-1 (GLP-1) secreted from distal small intestine. They are known to regulate insulin secretion, thus postprandial glucose levels are appropriately maintained [11]. GLP-1 is secreted in response to nutrient ingestion. In addition to the promotion of insulin secretion, GLP-1 possesses various physiological functions including suppression of glucagon secretion and suppression of appetite. GLP-1 receptor (GLP-1R) is the principal target of GLP-1 and is localized at the cellular surface. Recently various GLP-1 analogs are invented and rapidly becoming a primary glycemic control agent in type 2 diabetes [12]. These agents significantly improve blood glucose control and promote weight loss, but the risk of hypoglycemia accompanying the function is reported to be low. In addition to this, several pleiotropic functions of GLP-1R agonists have been suggested because GLP-1R is detected in the brain, lung, pancreatic islets, stomach, hypothalamus, heart, intestine, ovary, and endometrium [13–15]. Moreover, a previous report suggested that exenatide, a GLP-1R agonist, induced apoptosis in endometrial cancer cells [14]. Other studies have shown that exenatide is associated with autophagy induction [16, 17] and inhibits tumorigenicity and metastasis in human pancreatic cancer cells via the PI3K/AKT pathway [18]. However, the pathophysiological role of GLP-1R in endometrial cancer has not been fully elucidated.

Herein, we investigated the effects of the GLP-1R agonist liraglutide in endometrial cancer cells because the effects of liraglutide in endometrial cancer is relatively poorly studied compared to those of exenatide and examined the association between GLP-1R expression and clinicopathological characteristics in endometrial cancer patients.

## Methods

### Cell culture and chemicals reagents

The Ishikawa cells were obtained from Dr. Katsutoshi Oda (The University of Tokyo) and cultured in Dulbecco’s Modified Eagle Medium (DMEM, Gibco, Grand Island, NY, USA) containing 10% fetal bovine serum (Gibco), and maintained at 37 °C in 5% CO_2_. Liraglutide was purchased from Novo Nordisk (Bagsværd, Denmark), and AICAR was purchased from WAKO chemicals (Osaka, Japan). Mouse monoclonal antibody anti-β-actin (sc-47778, Santa Cruz Biotechnology, Dallas, TX, USA), anti-LC3 (M152–3, MBL, Nagoya, Japan), anti-p62 (M162–3, MBL), anti-p53 (DO-7, M7001, DAKO, Glostrup, Denmark), anti-estrogen receptor (ER; 1D5, M7047, DAKO), and anti-progesterone (PR; PgR636, M3569, DAKO) were purchased and used for this study. Rabbit polyclonal antibody against GLP-1R (bs-1559R) was purchased from Bioss ANTIBODIES (Boston, MA, USA). Rabbit monoclonal antibodies against AMPK (5831) and p-AMPK (4188) were purchased from Cell Signaling Technology (Danvers, MA, USA). Horseradish peroxidase (HRP)-conjugated donkey anti-rabbit IgG and sheep anti-mouse IgG secondary antibodies for Western blotting were purchased from GE Healthcare (Little Chalfont, Buckingamshire, UK), and the FITC-conjugated donkey anti-mouse IgG antibody for immunofluorescence was purchased from Santa Cruz.

### Cellular viability assay

Ishikawa cells at a density of 3 × 10^3^/well were seeded in 96-well microplates. After attachment, the cells were serum-starved for 12 h and incubated with liraglutide (0, 10, 100, and 1000 nmol/l) for 96 h. To measure the cellular viability, 10 μl of tetrazolium salt WST-8 (Cell Counting Kit-8; Dojindo, Tokyo, Japan) was added, and the optical density of samples was quantified by measuring absorbance at 450 nm using a microplate reader (Bio Tek, Winooski, VT, USA). Cellular viability was expressed as percentage relative to cells treated with medium alone. All the experiments were consisted of triplicate wells for each sample, and were repeated three times.

### Colony formation assay

Prior to the assay, cells at a density of 3 × 10^3^/well were seeded in 6-well plates. After attachment of the cells, they were serum-starved for 12 h and then incubated in the presence of liraglutide (0, 10, 100, and 1000 nmol/l), and the cells were allowed to grow for 10 days. Thereafter, the supernatant was discarded and the cells were fixed and stained with 0.5% crystal violet (Sigma-Aldrich) for 10 min, and the number of colonies was counted. All the experiments, each consisting of triplicate wells for each sample, were repeated three times, and representative images are shown.

### Western blotting

The cells were lysed and soluble protein was extracted. After measurement of protein concentration, equal amounts of proteins were loaded and separated by SDS-PAGE and transferred onto a polyvinylidene difluoride membrane (Millipore, Bedford, MA, USA). The membranes were blocked, incubated with appropriate primary antibodies, and were incubated with secondary antibodies. Signals were detected using an ImageQuant LAS 4000 Mini instrument (GE Healthcare, Wauwatosa, WI, USA). The amount of target proteins was internally normalized by ImageJ software (http://rsb.info.nih.gov/ij/). The experiments were repeated three times.

### Immunofluorescence

Ishikawa cells were cultured on Chamber Slides™ (Nunc, Rochester, NY, USA). After 12 h of incubation in serum-free DMEM, the medium was replaced with fresh DMEM containing serum and various different concentrations of liraglutide and AICAR. The cells were then incubated for 72 h and fixed in 4% paraformaldehyde. The cells were permeabilized, blocked in PBS containing 6% bovine serum albumin (BSA) for 30 min, and were incubated overnight with a primary antibody (1:200, anti-LC3) at 4 °C. The cells were further incubated with a secondary antibody (1:200, Alexa Fluor 488 Goat Anti-Mouse IgG) for 1 h at room temperature in the dark. The nuclei were counterstained with Hoechst 33342 (1:1000 dilution). The stained cells were visualized using a confocal fluorescence microscope (FV10i; Olympus, Japan). Representative results are shown.

### Flow cytometry analysis

Ishikawa cells seeded at a density of 5 × 10^4^/well were incubated with serum-free DMEM in 6-well plates. After 12 h, the media were replaced with DMEM containing serum and indicated concentration of liraglutide and the cells were further incubated for 96 h. For the analysis of cellular apoptosis, the harvested cells were washed extensively with PBS and were then stained using Annexin V and PI (Annexin V-FITC Apoptosis Detection Kit I; BD Biosciences, San Jose, CA, USA). The number of apoptotic cells defined as Annexin V / PI double positive cells was detected and analyzed (FACS Canto II; BD Biosciences). To analyze cell cycle distribution, the cells were stained with a BrdU Flow Kit (BD Biosciences) 96 h after exposure to liraglutide and flow cytometry analysis of the cells was performed. The experiments, each consisting of triplicate wells for each sample, were repeated three times.

### Immunohistochemistry

Tissue samples were formalin fixed, embedded in paraffin wax, and were cut into 4 μm. Paraffin sections were dewaxed in xylene and rehydrated through graded ethanol to water. Antigens were retrieved by boiling in 10 mM citriate buffer (pH 6.0) and endogenous peroxidase activity was quenched in methanol containing 3% hydrogen peroxide. The sections were incubated in PBS containing 3% BSA to block nonspecific binding, and were incubated for 30 min with primary antibodies including anti-GLP-1R (1:100), anti-p53 (1:50), anti-ER (1:50), and anti-PR (1:800). We tested normal endometrial tissue as a positive control, and negative control tissues were incubated without primary antibodies. The sections were subsequently incubated with secondary antibodies and Envision FLEX (DAKO). The antibody binding was visualized using a 3,30-diaminobenzidine solution (DAKO). After the sections were briefly counterstained with Mayer’s hematoxylin, the sections were dehydrated through a graded ethanol series and mounted.

### Patients and analysis of tumor samples specimens

We analyzed GLP-1R expression in 154 patients with endometrial cancer who underwent surgery at the Teikyo University Hospital from January 2003 to December 2012 using a tissue microarray (TMA). Clinical characteristics were obtained by a retrospective review of medical records (Additional file 1: Table S1). We classified G1 and G2 endometrioid cancer as low histological grade cancers, in contrast to G3 endometrioid cancer or serous, clear, or undifferentiated carcinoma, which were classified as high histological grade cancers. We defined diabetes mellitus (DM), hypertension (HT), and dyslipidemia (DL) as lifestyle diseases.

Two researchers blinded to patient background and clinical outcome independently reviewed the stained slides. GLP-1R and hormone receptors staining were evaluated according to the Allred score [19], a semi-quantitative system that takes into consideration the proportion of positive cells (scored on a scale of 0–5) and staining intensity (scored on a scale of 0–3). The proportion and intensity scores were added, yielding the Allred score (0–8). The cut-off level for GLP-1R was ≥6, and that for hormone receptors was ≥3, which is the cut-off level usually used in the diagnosis of breast cancer. We divided the samples into two groups, a high expression group and a low expression group. A score ≥ 6 was taken to represent high GLP-1R expression. For hormone receptors, a score ≥ 3 was considered positive. Gene mutations were evaluated as described previously [20].

### Statistical analysis

The graph bars are presented as the mean ± standard error (SE). Statistical significance was determined using Student’s t-test or one-way ANOVA with Turkey’s post hoc test using the GraphPad Prism 6 software (GraphPad, San Diego, CA) and JMP 10 (SAS Institute, Tokyo, Japan) as appropriate. The relationship between GLP-1R expression status and demographic data was analyzed by Pearson’s χ^2^ test. Survival rate of patients was calculated using Kaplan–Meier methods and differences were analyzed by log-rank test. A *p* value less than 0.05 was considered statistically significant.

## Results

### Liraglutide inhibits cancer cell growth in a dose-dependent manner

**Fig. 1:**
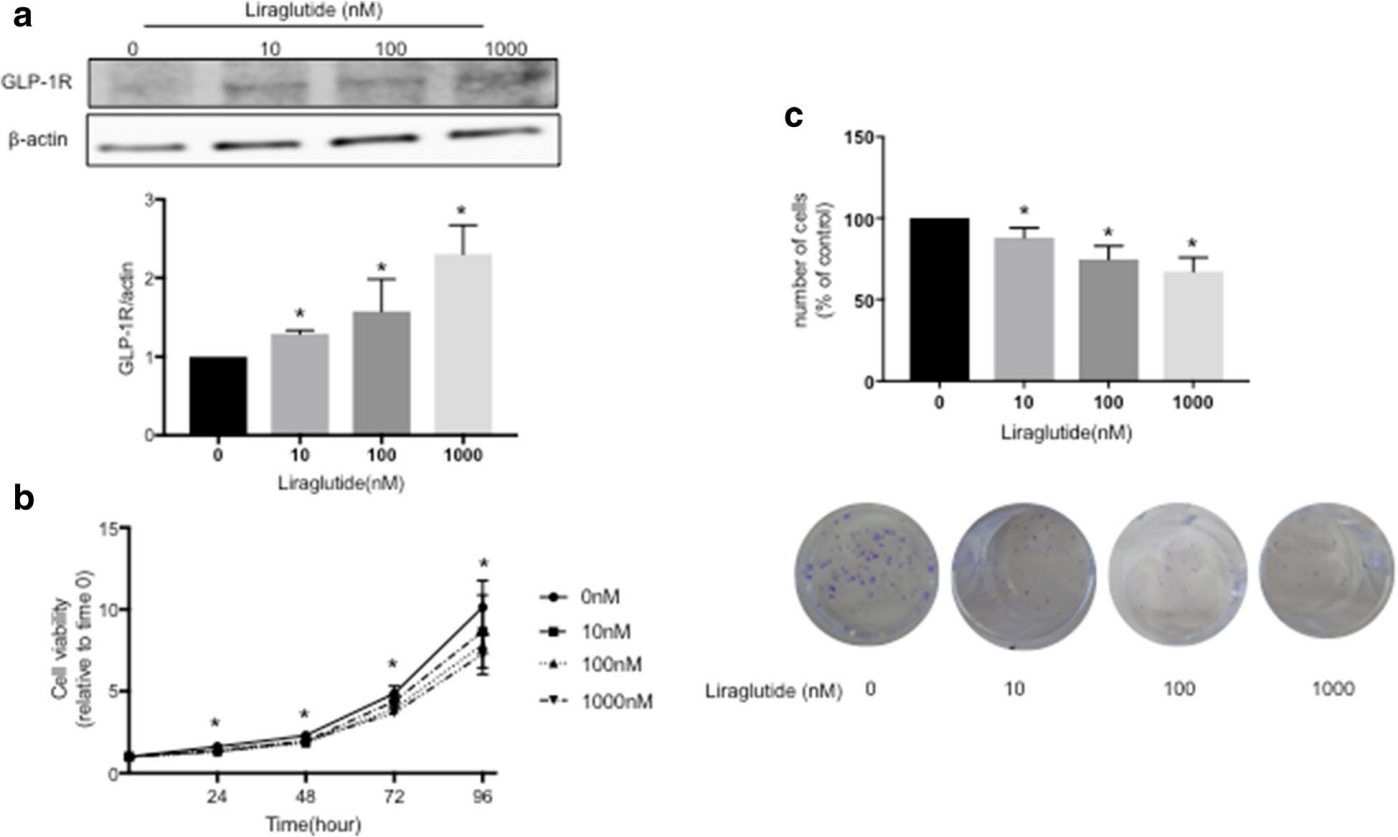
GLP-1R expression in Ishikawa endometrial cancer cells, and inhibition of cell viability and colony formation by liraglutide. **a** Ishikawa cells were harvested, and GLP-1R expression was determined by Western blot analysis. A representative Western blot result is shown. We found that liraglutide dose-dependently increased GLP-1R expression in Ishikawa cells. * denotes means significantly different from control (*p* <  0.05; ANOVA). **b** Cell viability was evaluated by an MTT assay. The viability of Ishikawa cells treated with 10, 100 and 1000 nM liraglutide was significantly lower at 24, 48, 72 and 96 h than that of untreated cells (0 nM). * denotes means significantly different from control (*p* <  0.05; ANOVA). **c** A colony formation assay revealed that the number of colonies was significantly decreased in cells treated with liraglutide compared with control cells. Images of representative clones are shown with the bar graph. * denotes means significantly different from control (p <  0.05; ANOVA)

We first investigated whether endometrial cancer cells express GLP-1R by Western blot analysis. We incubated Ishikawa cells with different concentrations of the GLP-1R agonist liraglutide for 96 h. The dose of liraglutide was empirically and preliminary investigated using control, 10 nM, 100 nM and 1000 nM considering the previous report [18]. Liraglutide dose-dependently increased GLP-1R expression in Ishikawa cells (Fig. 1a). To further investigate the effect of liraglutide, we performed cell viability and colony formation assays. The viability of Ishikawa cells treated with 10, 100 and 1000 nM liraglutide was significantly lower at 24, 48, 72 and 96 h than that of untreated cells (0 nM) (Fig. 1b). In addition, the number of colonies was significantly decreased in cells treated with liraglutide compared with control cells (Fig. 1c). In accordance with previous reports, liraglutide inhibited cancer cell growth in a dose-dependent manner.

### Liraglutide stimulates autophagy via the AMPK signaling pathway

**Fig. 2:**
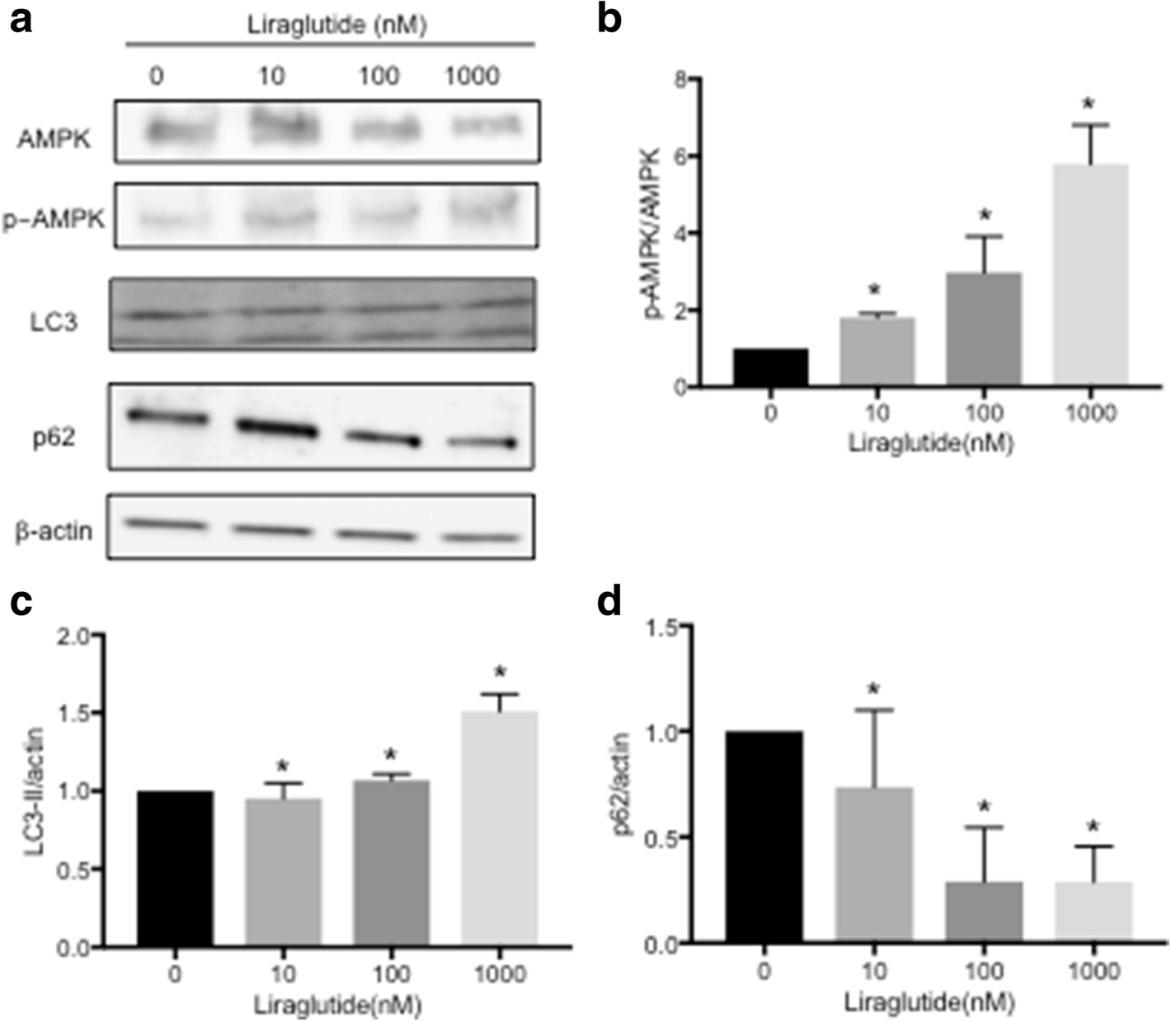
Status of AMPK phosphorylation and marker of autophagy expression in Ishikawa endometrial cancer cells treated by liraglutide. **a** Ishikawa cells were treated with different concentrations of liraglutide for 96 h. The cells were harvested, and AMPK, p-AMPK, LC3-II and p62 expression was analyzed by Western blot. β-actin was used as an internal control. Representative Western blot results are shown. LC3-II is defined as the lower band in the panel. **b** Quantification of the p-AMPK/AMPK ratio. * denotes means significantly different from control (*p* <  0.05; ANOVA). **c** Quantification of the LC3-II/β-actin ratio. * denotes means significantly different from control (p <  0.05; ANOVA). **d** Quantification of the p62/β-actin ratio. * denotes means significantly different from control (*p* <  0.05; ANOVA)

**Fig. 3:**
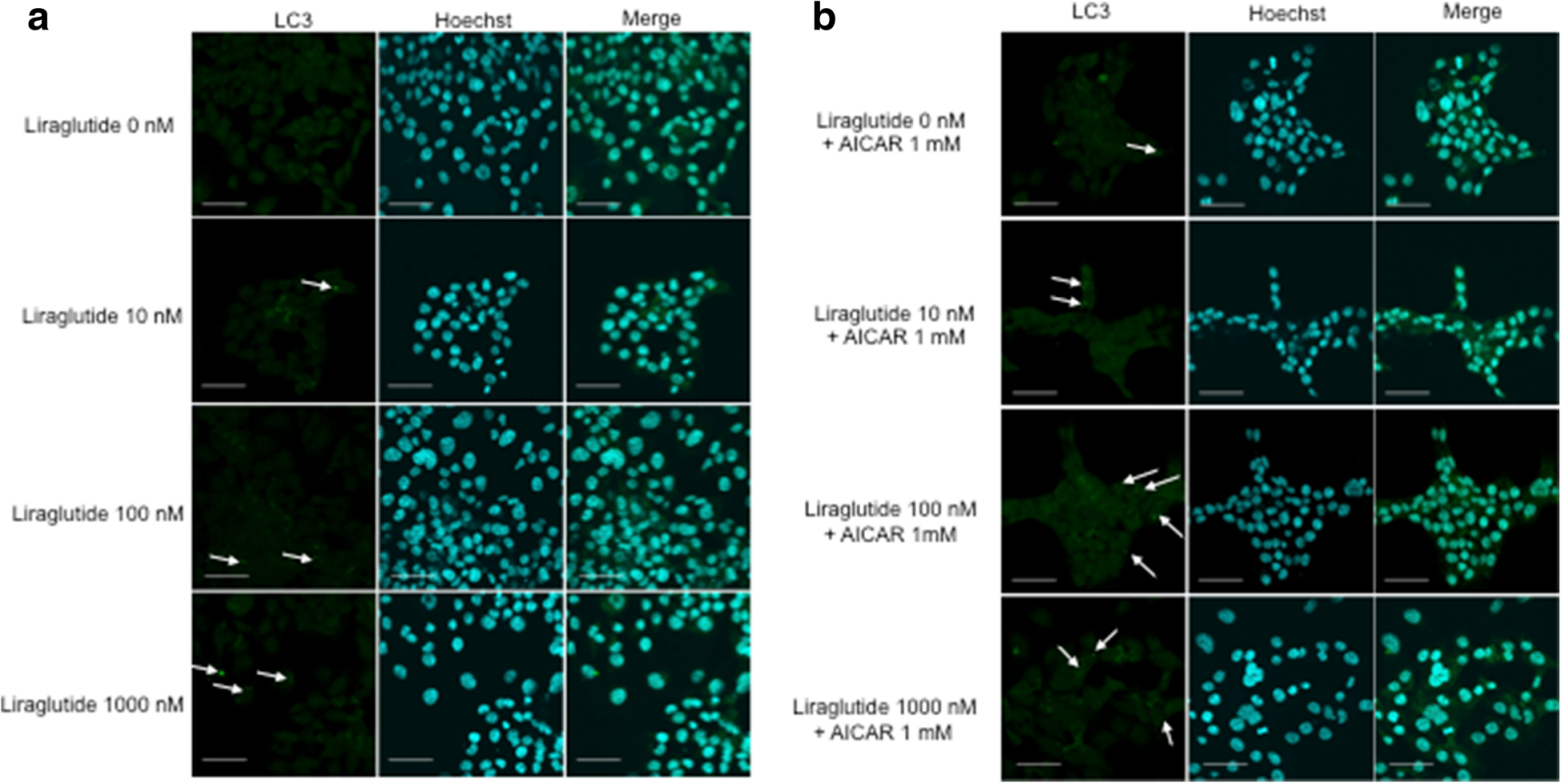
LC3 expression in Ishikawa cells and autophagosome accumulation by liraglutide. **a** Immunofluorescence staining of LC3 in Ishikawa cells treated with liraglutide for 72 h. Representative images from three independent experiments are shown. Ishikawa cells were treated with different concentrations of liraglutide. Autophagosome accumulation was evident in liraglutide-treated cells compared with control cells. **b** Ishikawa cells were treated with liraglutide and AICAR (1.0 mM) for 72 h. The combination of liraglutide and AICAR further promoted autophagosome accumulation. Nuclei were counterstained with Hoechst 33342. The small green dots (indicated by arrows) represent autophagosomes. Scale bar = 50 μm

To determine whether liraglutide stimulates autophagy via the AMPK signaling pathway, Ishikawa cells were treated with different concentrations of liraglutide for 96 h, and AMPK, p-AMPK, LC3 and p62 expression was analyzed by Western blot. AMPK and p-AMPK expression increased in a dose-dependent manner (Fig. 2). The protein levels of LC3 and p62 positively and negatively correlate with autophagy, respectively. This study showed that liraglutide significantly induced LC3 expression and decreased p62 expression in a dose-dependent manner (Fig. 2). Taken together, these results demonstrated that liraglutide stimulates autophagy via the AMPK pathway. Moreover, we performed immunofluorescence analysis using a monoclonal LC3 antibody to assess autophagosome accumulation after the addition of liraglutide and/or AICAR, an AMPK activator. Though it was difficult to quantify, immunofluorescence staining showed that autophagosome accumulation increased in liraglutide-treated cells compared with control cells (Fig. 3a). To confirm the role of the AMPK pathway, AICAR was added together with liraglutide, and liraglutide plus AICAR further upregulated autophagosome accumulation compared with liraglutide alone (Fig. 3b).

### Liraglutide induces apoptosis via the AMPK signaling pathway

**Fig. 4:**
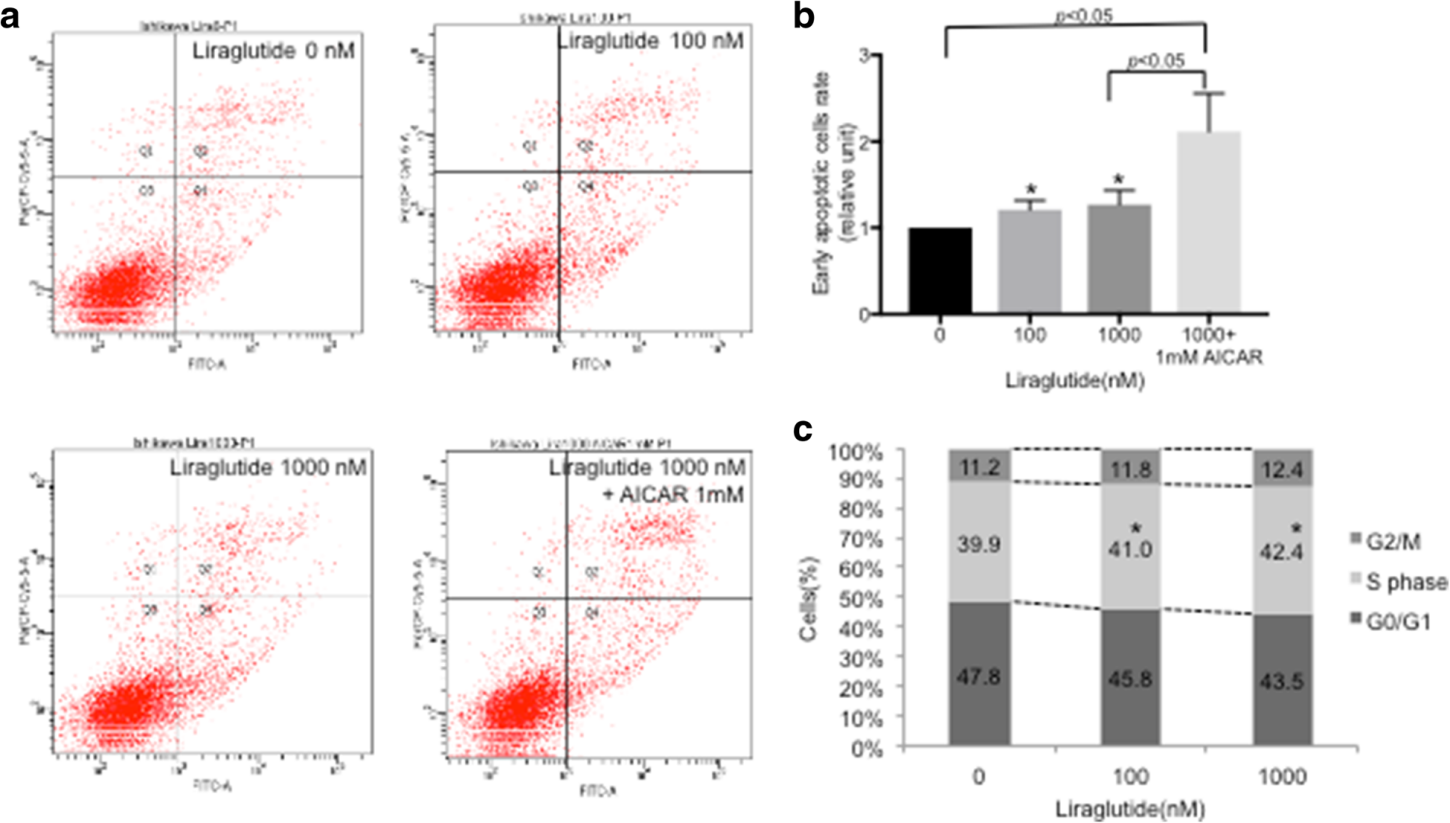
The effect of liraglutide on Ishikawa cell apoptosis measured by flow cytometry. **a** Apoptosis was detected by annexin-V and PI double staining. The proportion of early apoptotic cells increased after liraglutide treatment in a dose-dependent manner compared with control cells. A representative apoptosis analysis result is shown. **b** The results of three independent experiments were quantified. Our results demonstrated that the proportion of early apoptotic cells increased after liraglutide treatment in a dose-dependent manner compared with control cells (* *p* <  0.05; ANOVA). Comparison between control cells and liraglutide (1000 nM) + 1 mM AICAR, and between liraglutide (1000 nM) and liraglutide (1000 nM) + 1 mM AICAR were statistically significant (* p <  0.05; t-test). **c** We also found that the proportion of cells arrested in S phase was significantly higher in cells treated with liraglutide than in control cells. The results are expressed as the percentage of cells in each phase of the cell cycle (G2/M, S, G0/G1). The results are shown as the mean of three independent experiments (* *p* < 0.05; t-test)

We next investigated the mechanism underlying liraglutide-induced growth inhibition and mediation of the AMPK signaling pathway in Ishikawa cells using flow cytometry. The proportion of early apoptotic cells increased upon liraglutide treatment in a dose-dependent manner compared with control cells (Fig. 4a and b). In addition, the proportion of early apoptotic cells was higher upon treatment with liraglutide (1000 nM) plus AICAR (1 nM) than with liraglutide (1000 nM) alone (Fig. 4a and b). Further analysis showed that the proportion of cells arrested in S phase was significantly higher in liraglutide-treated cells than in control cells (Fig. 4c).

### Evaluation of GLP-1R expression in endometrial cancer

**Fig. 5:**
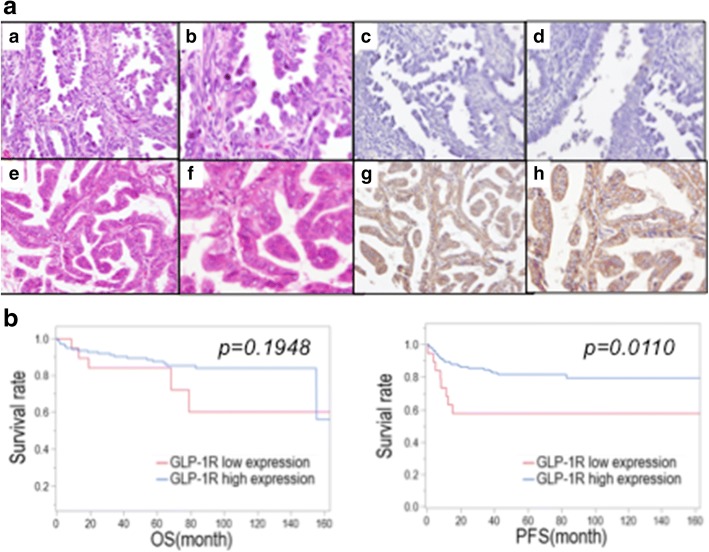
Histopathological presentation of endometrial cancer. **a** A representative case of low GLP-1R expression (Allred score 2). Hematoxylin and eosin staining, original magnification X200 (*a*) and X400 (*b*), and immunohistochemical staining of GLP-1R, original magnification X200 (*c*) and X400 (*d*), are shown. (*e*-*h*) A representative case of high GLP-1R expression (Allred score 8). Hematoxylin and eosin staining, original magnification X200 (*e*) and X400 (*f*), and immunohistochemical staining of GLP-1R, original magnification X200 (*g*) and X400 (*h*), are shown. **b** Kaplan–Meier survival curves for patients with endometrial cancer according to the degree of GLP-1R expression. The higher expression of GLP-1R was significantly correlated with better progression-free survival (right panel) (* *p* < 0.05; log-rank test). OS: overall survival, PFS: progression-free survival

GLP-1R expression was evaluated in endometrial cancer tissue samples from 154 patients based on the Allred score. Figure 5 shows the representative results of tissues with low (Fig. 5A, c and d) and high (Fig. 5A, g and h) GLP-1R expression. GLP-1R was predominantly localized in the cytoplasm in endometrial cancer tissue. Low expression and high expression were observed in 12.3% (19/154) and 87.7% (135/154) of patients, respectively, and 52.6% (10/19) of patients with low GLP-1R expression had no GLP-1R expression.

### Patients with high GLP-1R expression have better progression-free survival

We analyzed survival in endometrial cancer patients using the Kaplan–Meier method (Fig. 5B). We found that 25 patients died due to the disease, and 32 patients experienced disease recurrence. First, we assessed the association between GLP-1R expression and overall survival (OS) and progression-free survival (PFS).

High GLP-1R expression was significantly correlated with longer PFS. There was no statistically significant difference in OS between the two groups.

### Association between GLP-1R expression and clinicopathological characteristics in patients with endometrial cancer

GLP-1R expression was significantly associated with the differentiation of endometrioid carcinoma, histological grade, and ER and PgR status, although we did not find a significant association with obesity, DM or mutations in genes including PTEN and p53 (Table 1). The associations between high GLP-1R expression and histological grade, hormone receptor status and gene mutations were examined, and GLP-1R was highly expressed in 90.8% of patients with a low histological grade and 76.4% of patients with a high histological grade. ER and PgR were highly expressed in 93.8 and 83.3% of patients with a low histological grade, respectively. Meanwhile, there was no association between PTEN mutations and histological grade, but p53 expression was correlated with histological grade. In addition, more than 50% of patients with a high histological grade had a PTEN mutation (Table 2).

**Table 1:** The immunohistological analysis of GLP-1R revealed that the expression of GLP-1R was associated with positive estrogen and progesterone receptor status, and higher expression of GLP-1R was significantly correlated with better progression-free survival: relationship between GLP-1R expression status and clinicopathological characters

	GLP-1R expression			p
	Number	Low	High	
	154	19	135	
Age				
<50	41	4 (21.0%)	37 (27.4%)	NS
≧50	113	15 (79.0%)	98 (72.6%)	
Histological grade				
low*	120	11 (57.8%)	109(90.8%)	0.0367
high**	34	8 (42.1%)	26 (76.4%)	
FIGO stage				
Early (stage I-II)	113	14 (73.6%)	99 (73.4%)	NS
Advanced (stage III-IV)	41	5 (26.4%)	36 (26.6%)	
Myometrial invasion				
< 1/2	92	11 (57.8%)	81 (60.0%)	NS
≧1/2	62	8 (42.2%)	54 (40.0%)	
Lymph node metastasis				
pN0	125	14 (73.7%)	111(82.2%)	NS
pN1	29	5 (26.3%)	24 (17.8%)	
Distant metastasis				
M0	143	18 (94.7%)	125(92.6%)	NS
M1	11	1 (5.3%)	10 (7.4%)	
ER				
-	17	5 (27.7%)	12 (9.6%)	0.0271
+	125	13 (72.3%)	112(90.4%)	
PgR				
-	32	8 (44.4%)	24 (17.9%)	0.0095
+	120	10 (55.6%)	110(82.1%)	
BMI (kg/m^2^)				
< 25	80	14 (77.8%)	66 (54.1%)	NS
≧ 25	60	4 (22.2%)	56 (45.9%)	
DM				
+	122	14 (77.7%)	108(81.8%)	NS
-	28	4 (22.3%)	24 (18.2%)	
Hypertension				
+	47	6 (12.7%)	41 (87.3%)	NS
-	103	12 (11.6%)	91 (88.4%)	
Dyslipidemia				
+	13	3 (23.1%)	10 (76.9%)	NS
-	137	15 (11.0%)	122(89.0%)	
PTEN mutation				
-	62	11 (61.1%)	51 (38.6%)	NS
+	88	7 (38.9%)	81 (61.4%)	
p53 mutation				
-	128	15 (78.9%)	113(83.7%)	NS
+	26	4 (21.1%)	22 (16.3%)	

*histological low grade cancers: G1 and G2 endometrioid cancer

**histological high grade cancers: G3 endometrioid cancer or serous, clear, or undifferentiated carcinoma

**Table 2:** The immunohistological analysis of GLP-1R revealed that the expression of GLP-1R was associated with positive estrogen and progesterone receptor status, and higher expression of GLP-1R was significantly correlated with better progression-free survival: relationship between histological grade and GLP-1R

	GLP-1R high expression	ER positive	PgR positive	PTEN mutation	p53 mutation
Low grade	90.8%(109/120)	93.8%(106/113)	88.3%(106/120)	59.8%(70/117)	8.3%(10/120)
High grade	76.4%(26/34)	65.5%(12/29)	43.7%(14/32)	54.5%(18/33)	47%(16/34)
p	< 0.05	< 0.001	< 0.001	NS	< 0.001

## Discussion

A recent study suggested that the GLP-1R agonist exenatide promoted apoptosis in Ishikawa cells and attenuated xenograft tumor growth in a mouse model [14]. They further showed that all endometrial cancer tissues expressed GLP-1R, but the mechanism of GLP-1R-induced apoptosis and the association between clinicopathological characteristics of endometrial cancer patients and GLP-1R expression remains to be elucidated. Here, we investigated the physiological effect of liraglutide, a GLP-1R agonist, in Ishikawa endometrial cancer cells, and the pathological roles of GLP-1R in patients with endometrial cancer were analyzed using tissue samples.

First, our results showed that GLP-1R expression was elevated by liraglutide in a dose-dependent manner in Ishikawa endometrial cancer cells. This result agrees with a previous study using liraglutide in pancreatic cancer cells [18]. To investigate whether liraglutide inhibits endometrial cancer cell proliferation, we performed cell viability and colony formation assays and found that liraglutide suppresses the growth of Ishikawa cells in a dose-dependent manner, in accordance with a previous report [14]. This result is not surprising, because the GLP-1R agonist exenatide has been shown to inhibit the growth of colon, prostate and breast cancer cells [14]. It has been shown that metformin, a biguanide compound primarily used for the treatment of DM, has pleiotropic functions and can inhibit cell growth in a number of cancer types; thus, metformin has chemo-preventive and anti-proliferative properties. Adjuvant metformin treatment could improve the relapse-free survival rate in patients with stage IA endometrial cancer, and metformin use was associated with improved recurrence-free survival and OS; in addition, metformin enhanced apoptosis induced by paclitaxel and cisplatin [21, 22]. We propose that liraglutide may also be useful as an adjuvant therapy in endometrial cancer.

Moreover, our results showed that liraglutide could activate the AMPK signaling pathway, a master regulator of energy homeostasis, and induces autophagy in a dose-dependent manner, as demonstrated by Western blotting (Fig. 2). We confirmed that liraglutide induced autophagosome accumulation, and the combination of liraglutide and AICAR, an AMPK activator, enhanced autophagosome accumulation compared with liraglutide alone (Fig. 3). Thus, our study suggests that liraglutide induces autophagy via the AMPK signaling pathway. This result is not surprising, because exenatide also regulates the AMPK signaling pathway to promote apoptosis in endometrial cancer cells [14]. Meanwhile, metformin is also an AMPK activator and can activate the autophagy pathway [23] by inducing AMPK phosphorylation [24]. The AMPK signaling pathway has been demonstrated to be a positive regulator of autophagy [23], and AMPK acts as a metabolic checkpoint in the metabolic monitoring system and stimulates autophagy [25]. Our results thus clearly demonstrated the mechanism of a GLP-1R agonist in endometrial cancer cells.

To further elucidate the mechanism underlying the reduction in cell viability shown in Fig. 1, we performed flow cytometry. Liraglutide upregulated the rate of early apoptosis in a dose-dependent manner, and liraglutide plus AICAR further upregulated the rate of early apoptosis (Fig. 4). In addition, liraglutide arrested Ishikawa cells in S phase, indicating that significant effect of liraglutide, as cells arrested in S phase are prone to die due to activated apoptosis signaling [26]. Programmed cell death can be divided into two categories, namely, apoptosis (type I cell death) and autophagy (type II cell death), and autophagy acts as a double-edged sword; it is primarily a protective process for cells but can also play an important role in cell death [27]. Thus, we conclude that liraglutide simultaneously induced type I and type II cell death. However, the role of autophagy in cancer is controversial. Autophagy is a physiological cellular process, and it can suppress carcinogenesis by eliminating oncogenic molecules and damaged organelles [28]. Conversely, after the establishment of invasive cancer, autophagy and intracellular recycling of degraded metabolites can be disrupted, which might promote tumor growth [28]. Thus, it should be noted that the effects of autophagy on cancer cells could depend on cellular characteristics, the genetic background of patients, and the tumor microenvironment [29], but manipulating the autophagy pathway to affect cancer progression is a popular research area, and it has the potential to be a novel therapeutic strategy [30–32]. The role of autophagy in regulating cancer cell fate remains controversial, and further investigations are warranted.

Then the associations were analyzed between immunohistochemical staining of GLP-1R and clinical characteristics. Endometrial cancer tissues also expressed GLP-1R, and high GLP-1R expression was clearly associated with better PFS (Fig. 5). Moreover, we found that high GLP-1R expression was significantly associated with a positive hormone receptor status and low histological grade (Tables 1 and 2). ER- and/or PgR-positive status is generally associated with better prognosis or survival of endometrial cancer, and hormone-receptor-negative status is considered an indicator of aggressive tumor growth and poor prognosis [33, 34]. Our data suggested that endometrial cancer patients with high GLP-1R expression tend to have type I endometrial cancer, based on the association of GLP-1R expression with hormone receptor status and low histological grade, but the demographic data failed to show a significant difference between GLP-1R status and BMI or lifestyle diseases, including DM, HT and HL.

High GLP-1R expression was associated with type I endometrial cancer, while it was not significantly associated with PTEN mutation status. Type I endometrial cancer exhibits a low histological grade, while type II exhibits a high histological grade. Type I endometrial cancer typically involves mutations in PTEN, K-ras and β-catenin, and type II often involves p53 mutations [35]. In other words, there is an association between low histological grade and PTEN mutations. However, the present study showed that 54.5% of patients with a high histological grade had PTEN mutations, contrary to the previous report [35]. It can be difficult to classify endometrial cancer into only two groups considering the gene mutations described above. Another recent study proposed a molecular pathological classification system of endometrial cancer [36] and identified 4 subtypes. Therefore, the molecular pathological classification of endometrial cancer is still in progress, and further investigation might provide an opportunity to integrate a genome-based classification system with molecular pathological findings in endometrial cancer.

This study had some limitations. First, we did not evaluate normal endometrial tissue by immunohistochemistry. It is important to investigate GLP-1R expression in normal endometrial tissue, as its expression could vary throughout menstrual cycle. Second, we did not study whether the combination of liraglutide and other drugs exerts anti-tumorigenic effects in endometrial cancer cells. Finally, we did not study the effect of liraglutide in vivo, and this should be investigated in the future.

## Conclusions

In this study, we shed light on the pathophysiological role of GLP-1R in endometrial cancer. Liraglutide induced apoptosis and autophagy via the AMPK signaling pathway, and GLP-1R may be a biomarker of endometrial cancer, as higher GLP-1R expression was be associated with better prognosis in endometrial cancer patients. Considering that metformin has been associated with a decrease in the incidence of cancer, the use of liraglutide to target autophagy in endometrial cancer cells may be a novel potential strategy for endometrial cancer treatment.

## Additional file

Additional file 1: **Table S1.** Detailed data of participants. Clinicopathological data of 154 patients with endometrial cancer who underwent surgery at the Teikyo University Hospital. These clinical characteristics were obtained by a retrospective review of medical records and the pathological data were obtained by our tissue microarray. (XLSX 14 kb)

## Abbreviations

AMPK: AMP-activated protein kinase
DL: Dyslipidemia
DM: Diabetes mellitus
ER: Estrogen receptor
GLP-1R: Glucagon-like peptide-1 receptor
HT: Hypertension
mTOR: Mammalian target of rapamycin
OS: Overall survival
PFS: Progression-free survival
PgR: Progesterone receptor
PI3K: Phosphatidylinositol-3 kinase
PTEN: Phosphatase and tensin homolog
TMA: Tissue microarray
